# Gram-Positive Bacterial Lipoglycans Based on a Glycosylated Diacylglycerol Lipid Anchor Are Microbe-Associated Molecular Patterns Recognized by TLR2

**DOI:** 10.1371/journal.pone.0081593

**Published:** 2013-11-21

**Authors:** Landry Blanc, Romain Castanier, Arun K. Mishra, Aurélie Ray, Gurdyal S. Besra, Iain Sutcliffe, Alain Vercellone, Jérôme Nigou

**Affiliations:** 1 CNRS; IPBS (Institut de Pharmacologie et de Biologie Structurale); 205 route de Narbonne, F-31077 Toulouse, France; 2 Université de Toulouse; UPS; IPBS; F-31077 Toulouse, France; 3 National Institute for Medical Research, London, United Kingdom; 4 School of Biosciences, University of Birmingham, Edgbaston, Birmingham, United Kingdom; 5 Faculty of Health and Life Sciences, Northumbria University, Newcastle upon Tyne, United Kingdom; Hopital Raymond Poincare - Universite Versailles St. Quentin, France

## Abstract

Innate immune recognition is the first line of host defense against invading microorganisms. It is a based on the detection, by pattern recognition receptors (PRRs), of invariant molecular signatures that are unique to microorganisms. TLR2 is a PRR that plays a major role in the detection of Gram-positive bacteria by recognizing cell envelope lipid-linked polymers, also called macroamphiphiles, such as lipoproteins, lipoteichoic acids and mycobacterial lipoglycans. These microbe-associated molecular patterns (MAMPs) display a structure based on a lipid anchor, being either an acylated cysteine, a glycosylated diacylglycerol or a mannosyl-phosphatidylinositol respectively, and having in common a diacylglyceryl moiety. A fourth class of macroamphiphile, namely lipoglycans, whose lipid anchor is made, as for lipoteichoic acids, of a glycosylated diacylglycerol unit rather than a mannosyl-phosphatidylinositol, is found in Gram-positive bacteria and produced by certain Actinobacteria, including *Micrococcus luteus*, *Stomatococcus mucilaginosus* and *Corynebacterium glutamicum*. We report here that these alternative lipoglycans are also recognized by TLR2 and that they stimulate TLR2-dependant cytokine production, including IL-8, TNF-α and IL-6, and cell surface co-stimulatory molecule CD40 expression by a human macrophage cell line. However, they differ by their co-receptor requirement and the magnitude of the innate immune response they elicit. *M. luteus* and *S. mucilaginosus* lipoglycans require TLR1 for recognition by TLR2 and induce stronger responses than *C. glutamicum* lipoglycan, sensing of which by TLR2 is dependent on TLR6. These results expand the repertoire of MAMPs recognized by TLR2 to lipoglycans based on a glycosylated diacylglycerol lipid anchor and reinforce the paradigm that macroamphiphiles based on such an anchor, including lipoteichoic acids and alternative lipoglycans, induce TLR2-dependant innate immune responses.

## Introduction

The innate immune system is genetically programmed to detect molecular signatures of microbes *via* a limited number of germline-encoded pattern recognition receptors (PRRs) [1], [2], [3], [4], [5], [6]. The signatures seen as foreign are structural motifs, referred to as pathogen/microbe-associated molecular patterns (PAMPs/MAMPs), which are unique to microorganisms and relatively invariant in most microorganisms of a given class. The molecular pattern recognized by PRRs is usually a small but conserved part of a macromolecule of microbial origin, such as the lipid anchor in bacterial macroamphiphiles, and which might be repeated such as monomers in peptidoglycan or in nucleic acids [7], [8].

**Figure 1:**
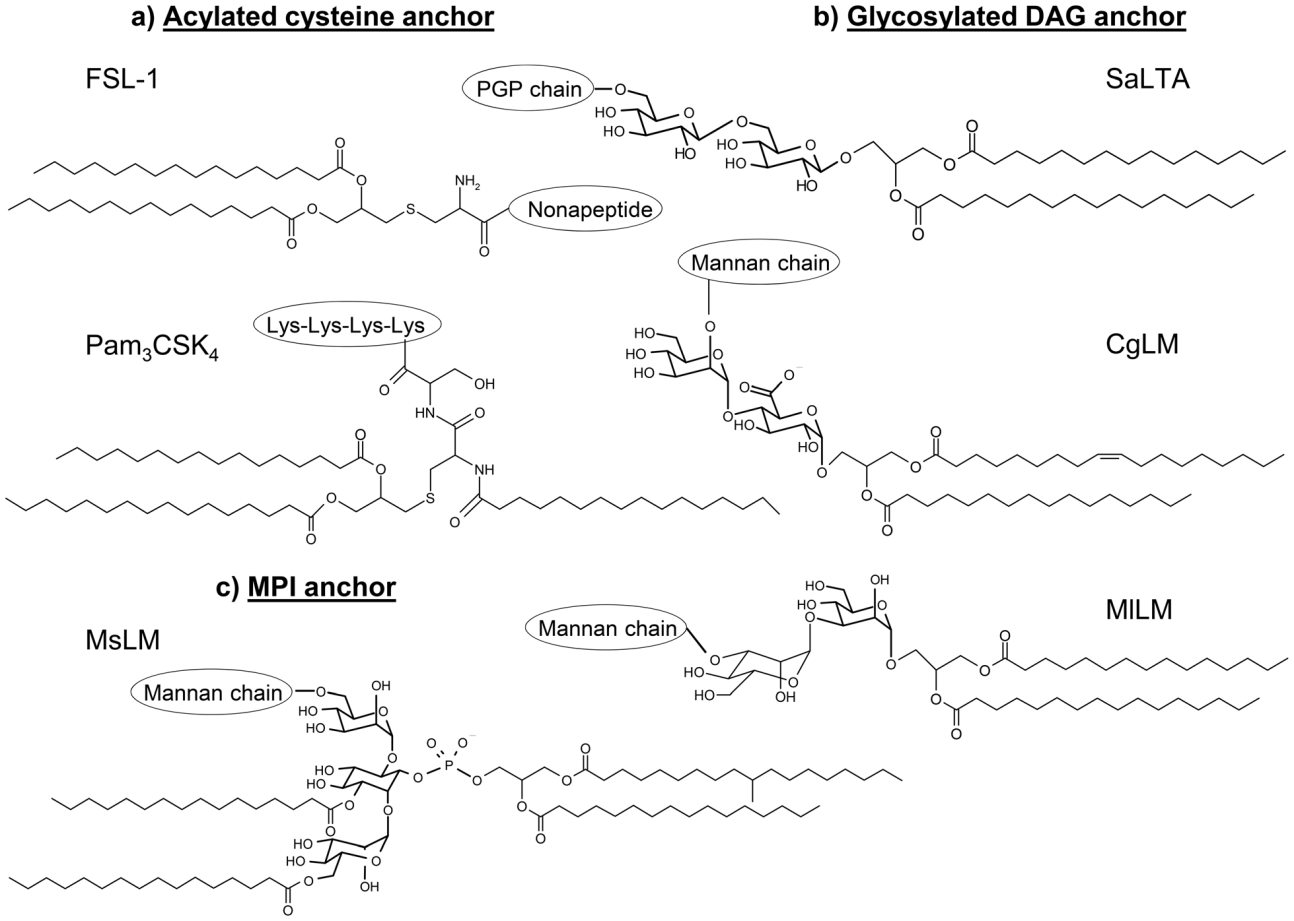
Structure of the lipid anchor of bacterial macroamphiphiles. CgLM, *Corynebacterium glutamicum* lipomannan; DAG, diacylglycerol; FSL-1, synthetic N-terminal part of lipopoprotein LP44 of *Mycoplasma salivarum*; MlLM, *Micrococcus luteus* lipomannan; MPI, mannosyl-phosphatidyl-*myo*-inositol; MsLM, *Mycobacterium smegmatis* lipomannan; PGP, polyglycerolphosphate; SaLTA, *Staphylococcus aureus* lipoteichoic acid; The available evidence [29] suggests the structure of *Stomatococcus mucilaginosus* lipomannan (SmLM) is very similar to that of MlLM.

Bacterial macroamphiphiles, i.e. cell envelope lipid-linked polymers [9], [10], namely Gram-negative bacterial lipopolysaccharide (LPS), Gram-positive bacteria lipoteichoic acid (LTA), lipoproteins and mycobacterial lipoglycans, evidently meet PAMP/MAMP criteria and are well suited to innate immune recognition. They are mostly recognized *via* their lipid anchor by a family of PRRs, named Toll-like receptors (TLRs). TLR extracellular domains, which have leucine-rich repeat modules, adopt a horseshoe-like shape structure and are responsible for PAMP/MAMP binding [11], [12]. Their intracellular signalling domains trigger innate immune responses through NF-κB-dependent and IFN regulatory factor-dependant signalling pathways [1], [2], [3], [4]. Lipoproteins, LTA and mycobacterial lipoglycans, based on a lipid anchor being either an acylated cysteine (a), a glycosylated diacylglycerol (b) or a mannosyl-phosphatidylinositol (c) respectively, and having in common a diacylglyceryl moiety (Figure 1), are recognized by TLR2. The latter generally functions as a heterodimer with either TLR1 or TLR6, which is involved in discrimination of the acylation state of lipoproteins, its best characterized agonists [13], [14], [15]. Structure/function relationship studies, corroborated by the reports of the structures of several TLR2-lipopeptide complexes determined by X-ray crystallography, have established that the acylated cysteinyl moiety is the structure recognized by the receptors and that triacylated lipoproteins are preferentially recognized by the TLR2/TLR1 complex, whereas diacylated lipoproteins are recognized by the TLR2/TLR6 complex [16], [17]. In the crystal structure of a TLR1-TLR2 heterodimer in complex with the model lipopeptide Pam_3_CSK_4_ [16] (Figure 1), the triacylated lipopeptide appears to form a bridge between TLR2 and TLR1 with the two ester-bound fatty acyl chains of the S-diacylglyceryl moiety inserted deep into a pocket in the hydrophobic core of TLR2, the third amide-linked acyl chain occupying a hydrophobic channel at the surface of TLR1 and the conserved polar head located at the region of contact between the two receptors. Crystal structure of a TLR2-TLR6-diacylated lipopeptide complex reveals that the lipid-binding channel of TLR6 is blocked by two phenylalanines that hamper the binding of any fatty acid chain [17]. Besides lipoproteins, the majority of low G+C Gram-positive bacteria (Firmicutes) produce LTA [10], [18], [19] that are composed of a lipid anchor, made of a diacylglycerol (DAG) unit substituted by a di- or tri-glycoside. In contrast, high G+C Gram-positive bacteria of the suborders *Corynebacterineae*, including mycobacteria, and *Pseudonocardineae* do not produce LTA but rather lipoglycans, with structures based on a multi-acylated mannosyl-phosphatidyl-*myo*-inositol (MPI) anchor [10], [19], [20], [21], [22], [23] (Figure 1). As described for diacylated lipopeptides, heterodimerization of TLR2 with TLR6 seems to be important for LTA recognition [24], [25]. A crystal structure of TLR2 in complex with *Streptococcus pneumoniae* LTA showed that the two fatty acyl chains of the DAG unit are inserted in the hydrophobic pocket of TLR2 [17]. Mycobacterial tri- and tetra-acylated lipoglycans are instead recognized by the TLR2-TLR1 heterodimer [26]. It is proposed, by analogy with lipopeptides, that in the case of tri-acylated lipoglycans, both fatty acids of the DAG unit are inserted in the hydrophobic pocket of TLR2 while the third acyl chain esterfying the mannosyl unit of the MPI anchor is inserted in the hydrophobic channel at the surface of TLR1 [8].

A fourth class of macroamphiphiles, namely lipoglycans whose lipid anchor is made, as for LTA, of a glycosylated DAG unit rather than a MPI, is found in some Gram-positive Actinobacteria, including *Micrococcus luteus* [27], [28], *Stomatococcus mucilaginosus* [29] and *Corynebacterium glutamicum* [30], [31] (Figure 1b). Here, we report that these alternative lipoglycans are also recognized by TLR2 and that they stimulate TLR2-dependant cytokine production and cell surface co-stimulatory molecule CD40 expression by a human macrophage cell line. Our results expand the repertoire of MAMPs recognized by TLR2 and reinforce the paradigm that macroamphiphiles based on a glycosylated DAG anchor, such as LTA and alternative lipoglycans, are ligands of TLR2.

## Results

Lipoglycans based on a glycosylated DAG anchor (i.e. DAG-based lipoglycans) were purified from *M. luteus* [27], [28], *C. glutamicum* [30] and *S. mucilaginosus* [29]. These lipoglycans are lipomannans (LM) (Figure 1) and will be subsequently termed MlLM, CgLM and SmLM respectively.

### Signaling via TLR2

**Figure 2:**
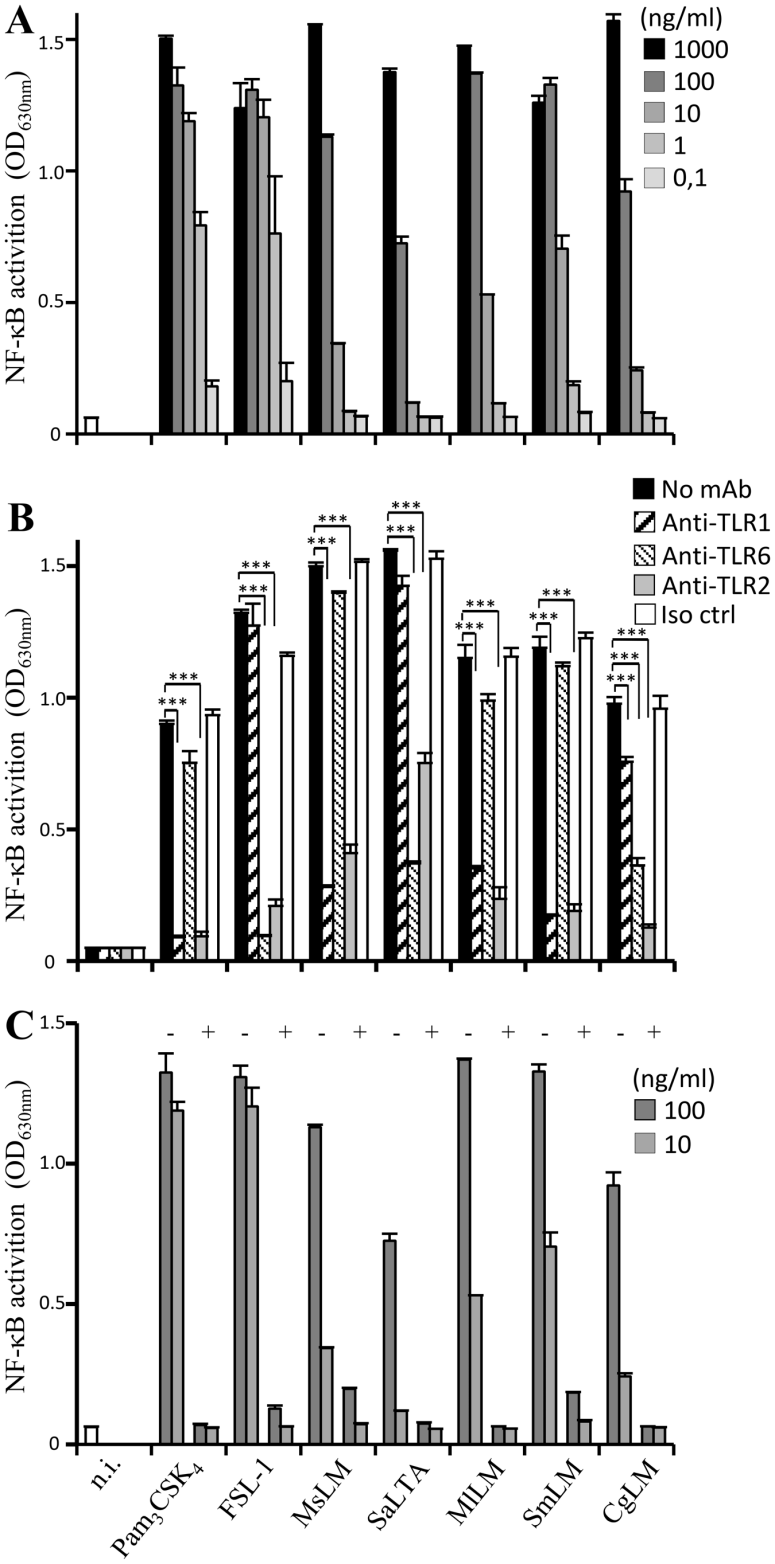
DAG-based lipoglycans are recognized by TLR2 in cooperation with TLR1 or TLR6. A, C) HEK-TLR2 cells were incubated with the various stimuli at a concentration ranging from 0.1 to 1000 ng/ml. B) HEK-TLR2 cells were pre-incubated or not for 30 min at 37°C before stimuli addition with various monoclonal antibodies: anti-TLR2 (5 µg/ml), anti-TLR1 (10 µg/ml), anti-TLR6 (10 µg/ml) or IgG1 isotype control (10 µg/ml). FSL-1 was tested at a concentration of 1 ng/ml, Pam_3_CSK_4_ and MsLM at 10 ng/ml, SmLM at 50 ng/ml, MlLM and SaLTA at 100 ng/ml and CgLM at 300 ng/ml. In C, deacylated molecules (+) were prepared by treating native molecules (−) with 2M NH_4_OH for 2h at 110°C. NF-κB activation was determined by reading OD at 630 nm. The results are mean ± SD of triplicate wells and are representative of at least three separate experiments. ***, P<0.001. Iso ctrl, isotype control, n.i., not induced.

We first tested the ability of MlLM, CgLM and SmLM to stimulate HEK293 cells stably transfected with human TLR2 and CD14 genes and a NF-κB-inducible reporter system (HEK-TLR2 cells). The synthetic lipopeptides Pam_3_CSK_4_ and FSL-1, *Mycobacterium smegmatis* LM (MsLM) and *Staphylococcus aureus* LTA (SaLTA) were used as reference ligands of TLR2 based on a lipid anchor being either an acylated cysteine, a MPI or a glycosylated DAG respectively (Figure 1). Interestingly, MlLM, SmLM and CgLM each induced NF-κB activation in a dose-dependent fashion (Figure 2A) and their activity was inhibited by an anti-TLR2 antibody (Figure 2B). However, the magnitude of activation varied between the different ligands of TLR2. Synthetic lipopeptides were the strongest agonists with an EC_50_ in the range of 1 ng/ml, followed by MlLM and SmLM (EC_50_ ∼ 10 ng/ml), MsLM (EC_50_ ∼ 30 ng/ml) and finally SaLTA and CgLM (EC_50_ ∼ 100 ng/ml).

TLR2 activation by MlLM, CgLM and SmLM was dependent on the presence of a native acylated lipid anchor since they failed to activate NF-κB upon deacylation (Figure 2C). This result is in agreement with structure/function relationships studies and crystal structures that previously revealed that fatty acids are critical for lipopeptides, LTA and mycobacterial lipoglycans signaling *via* and binding to TLR2 [7], [8], [16], [17]. The requirement of TLR1 or TLR6 for recognition of MlLM, CgLM and SmLM by TLR2 was tested using blocking antibodies. As expected for the reference ligands, the activity of the triacylated Pam_3_CSK_4_ and MsLM was clearly dependent on TLR1, whereas that of the diacylated FSL-1 lipopeptide and SaLTA was dependent on TLR6 (Figure 2B). The TLR2-dependant activation of NF-κB by CgLM was strongly, although not completely, inhibited by the anti-TLR6 antibody and moderately inhibited by anti-TLR1 antibody. Surprisingly, activity of diacylated MlLM and SmLM was almost completely inhibited by anti-TLR1 but not anti-TLR6 antibodies (Figure 2B).

### Activation of THP-1 human macrophage cell line

**Figure 3:**
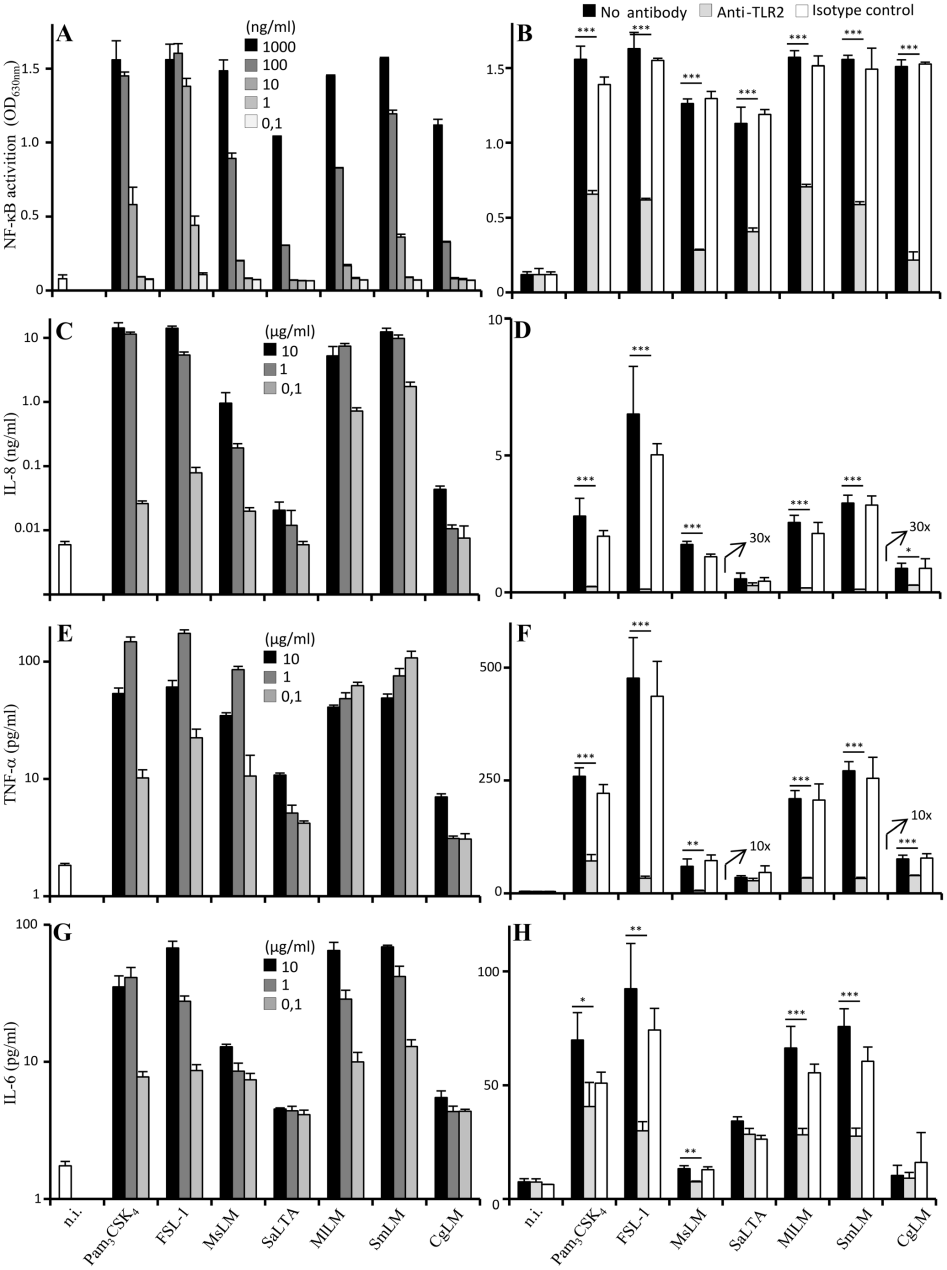
DAG-based lipoglycans stimulate TLR2-dependant NF-κB activation and IL-8, TNF-α and IL-6 production by human THP-1 monocyte/macrophage cell line. A) THP-1 cells were incubated with the various stimuli at a concentration ranging from 0.1 to 1000 ng/ml. C, E, G) THP-1 cells were incubated with the various stimuli at a concentration ranging from 0.1 to 10 µg/ml. B, D, F, H) THP-1 cells were pre-incubated or not for 30 min at 37°C with 10 µg/ml of anti-TLR2 or IgG1 isotype control antibodies and stimulated overnight with the different TLR2 ligands. FSL-1, Pam_3_CSK_4_ FSL-1, MsLM, MlLM, SmLM were tested at a concentration of 1 µg/ml, SaLTA and CgLM at 10 µg/ml. In A and B, NF-κB activation was determined by reading OD at 630 nm. In C to H, cytokines were assayed in the culture supernatant by sandwich ELISA. The results are mean ± SD of triplicate wells and are representative of at least three separate experiments. 10x and 30x mean that the concentration of cytokine detected is amplified 10 and 30 fold respectively. *, P<0.05; **, P<0.01; ***, P<0.001. n.i., not induced.

We then investigated the capacity of DAG-based lipoglycans to activate the human THP-1 monocyte/macrophage cells, using a cell line derivative that stably expresses a NF-κB-inducible reporter system. MlLM, SmLM and CgLM induced NF-κB activation in a dose-dependent fashion (Figure 3A) and their activity was strongly inhibited by an anti-TLR2 antibody (Figure 3B). The magnitude of activation of the different macroamphiphiles followed the hierarchy observed with HEK-TLR2 cells (Figure 2A). Lipopeptides were the most stimulatory with an EC_50_ below 10 ng/ml, followed by the TLR2-TLR1 signaling lipoglycans SmLM, MsLM and MlLM (EC_50_ below 100 ng/ml), and finally the TLR2-TLR6 signaling SaLTA and CgLM (EC_50_ above 300 ng/ml).

Activation of innate immunity ultimately regulates adaptive immunity, most particularly *via* the production of cytokines and the expression of co-stimulatory molecules by antigen-presenting cells [1], [2], [3], [4]. THP-1 cells being strong producers of IL-8 [32], this cytokine was analyzed first. All the macroamphiphiles induced a dose-dependent production of IL-8 (Figure 3C) that was almost completely abrogated by an anti-TLR2 antibody (Figure 3D). However, MlLM and SmLM were the most potent inducers, except for synthetic lipopeptides, a plateau of around 10 ng/ml of IL-8 being reached at a concentration of DAG-based lipoglycans of 1 µg/ml. SaLTA and CgLM were much weaker inducers, whereas MsLM showed an intermediate potency (Figure 3C). Similar data were obtained with TNF-α (Figure 3E) and IL-6 (Figure 3G), although these cytokines were produced by THP-1 cells in lower amount. It is worth noting that a TNF-α production plateau of around 100 pg/ml was reached for an MlLM or SmLM concentration of 100 ng/ml only (Figure 3E). An 1 log increase in concentration was required for MsLM to reach the same effect. A 2 log increase in concentration of SaLTA and CgLM led to a production of TNF-α of around 10 pg/ml only. TNF-α (Figure 3F) and IL-6 (Figure 3H) production elicited by the different stimuli was strongly and significantly inhibited by an anti-TLR2 antibody, except for SaLTA and CgLM (which induced insufficient amount of cytokines for accurate determination). These data confirm the earlier observation that CgLM stimulated TNF-α production by the human macrophage cell line THP-1 [31].

**Figure 4:**
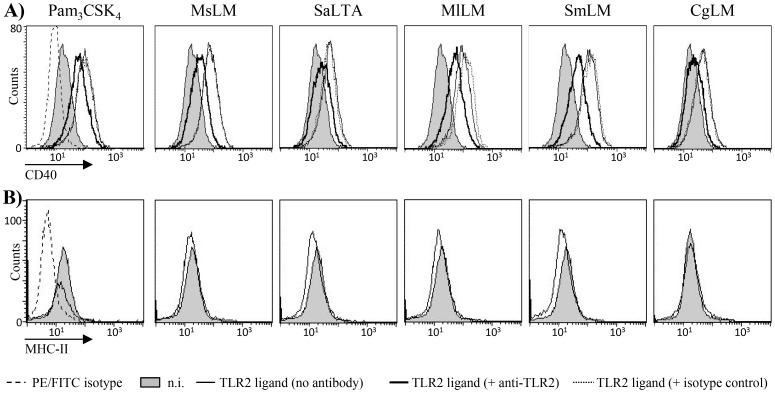
DAG-based lipoglycans stimulate TLR2-dependant cell surface co-stimulatory molecule CD40 but not MHCII expression by human THP-1 monocyte/macrophage cell line. THP-1 cells were pre-incubated or not for 30 min at 37°C with 10 µg/ml of anti-TLR2 or IgG1 isotype control antibodies and stimulated overnight with the different TLR2 ligands at a concentration of 1 µg/ml. CD40 (A) and MHC-II (B) expression was monitored by flow cytometry. One representative experiment (from three repeats) is shown. n.i., not induced.

Finally, all the TLR2 ligands tested were able, at a concentration of 1 µg/ml, to induce the expression of the cell surface co-stimulatory molecule CD40 but failed to induce MHCII (Figure 4). Again, DAG-based MlLM and SmLM were the most stimulatory lipoglycans. SaLTA and CgLM were the least stimulatory ligands whereas MsLM showed an intermediate potency. Induction of CD40 expression by the different stimuli was partially inhibited by an anti-TLR2 antibody (Figure 4).

## Discussion

Bacterial cell wall macroamphiphiles are detected by the innate immune system as foreign molecular signatures [8]. LPS is recognized by TLR4-MD2 whereas lipoproteins, LTA, and mycobacterial lipoglycans are recognized by TLR2-TLR1/TLR6. Structure/function relationships studies and crystal structures have established that macroamphiphiles are mostly recognized *via* their lipid anchor, most particularly through binding of fatty acids into hydrophobic pockets of the receptors [12]. Lipoproteins, LTA, and mycobacterial lipoglycans display a structure based on a lipid anchor consisting in an acylated cysteine, a glycosylated DAG or a MPI respectively. Besides these last three classes of macroamphiphiles, a fourth one is found in Gram-positive bacteria, namely lipoglycans whose lipid anchor is made of a glycosylated DAG unit.

We report here that representatives of these alternative DAG-based lipoglycans, MlLM, SmLM and CgLM, are also recognized by TLR2 and that they stimulate TLR2-dependant cytokine production, including IL-8, TNF-α and IL-6, and cell surface co-stimulatory molecule CD40 expression by a human macrophage cell line. However, they differ in their co-receptor requirement and the magnitude of the innate immune response they elicit.

As expected for a diacylated molecule, recognition of CgLM by TLR2 was mostly dependent on TLR6, although it was partially, but significantly, inhibited by an anti-TLR1 antibody. More surprisingly, MlLM and SmLM recognition was almost completely dependent on TLR1. These results are in sharp contrast with the paradigm of receptor usage as a function of the acylation pattern since triacylated lipoproteins are preferentially recognized by the TLR2/TLR1 complex, whereas diacylated lipoproteins are recognized by the TLR2/TLR6 complex [13], [14], [15]. However, it has been also previously described that in addition to the acylation pattern, the nature of the amino acids of the peptidic chain of lipopeptides can modulate the specificity of the recognition by TLR2 heterodimers [33], [34]. A similar phenomenon seems to apply with DAG-based lipoglycans, where the acylation pattern is not sufficient to dictate the TLR2 heterodimer usage. The underlying molecular bases remain to be uncovered, however these results further demonstrate that the pattern recognized by the receptors is not strictly restricted to the lipid moiety of lipopeptides or lipoglycans, but also involves in part the hydrophilic moiety of these TLR2 agonists. We previously established that the (α1→6)-mannopyranosyl backbone, which is a highly conserved structural feature of MPI-based lipoglycans, is an integral part of the pattern recognized by TLR2 [35]. Similarly, the mannan chain is required for DAG-based lipoglycan activity since DAG alone (dipalmitin) did not activate TLR2 (not shown).

Interestingly, SmLM and MlLM that induce signalling *via* TLR2-TLR1 were much stronger activators than CgLM that activates *via* TLR2-TLR6. Activity of SlLM and MlLM was even higher than that of the MPI-based lipoglycan MsLM, which contributes to the innate immune detection of *M. smegmatis* by TLR2-TLR1 [32]. In contrast, CgLM activity was weaker and similar to that of the TLR2-TLR6 agonist SaLTA. The weaker activity of CgLM and SaLTA was not a result of a low expression of TLR6 in THP-1 cells. Indeed, TLR6 expression was confirmed by flow cytometry analysis (not shown). Moreover, the TLR2-TLR6 ligand FSL-1 lipopeptide was highly stimulatory in these cells. An interesting correlate of the difference in response to CgLM compared to MlLM and SmLM is that the former has a distinctive charged α-glucopyranosyluronic residue proximal to the DAG anchor [30] rather than an uncharged mannosyl residue. Kang *et al.* [17] reported a crystal structure of TLR2 in complex with *S. pneumoniae* LTA, where the two fatty acyl chains of the DAG unit are inserted in the hydrophobic pocket of TLR2. Interestingly, although *S. pneumoniae* LTA was found by the authors to bind TLR2 with high affinity, in contrast to lipopeptides, it did not induce heterodimerization of the extracellular domains of TLR2 and TLR6. Although requiring TLR6 for activity, CgLM, similarly to *S. pneumoniae* LTA, may bind TLR6 with a weak affinity, and this might be correlated to the lower pro-inflammatory activity observed for LTA molecules [7] and CgLM as compared to lipopeptides. Nevertheless, the low affinity for TLR6 is most probably partly compensated in physiological conditions by the usage of soluble or membrane-anchored accessory receptors, such as CD14, LBP or CD36, that increase the bioavailability of the ligands [36], [37], [38]. As such, it is worth noting that cytokine production elicited by the different stimuli was investigated in the present study in non-differentiated THP-1 cells; in PMA-differentiated THP-1 cells, SaLTA was found to induce much higher concentrations of pro-inflammatory cytokines, although still less than the Pam_3_CSK_4_ lipopeptide [39].

In conclusion, our results expand the repertoire of MAMPs recognized by TLR2 to DAG-based lipoglycans and confirm the major role played by this receptor in the detection of Gram-positive bacteria [40]. Moreover, they reinforce the paradigm that macroamphiphiles based on a glycosylated DAG lipid anchor, such as LTA and alternative lipoglycans, induce TLR2-dependant innate immune responses. Although the diacylglyceryl unit, which is common to all the macroamphiphiles recognized by TLR2, is also found in lipids of higher eukaryotes, its substitution by a glycosylated polyglycerolphosphate chain in LTA, a (α1→6)-mannopyranosyl chain in alternative lipoglycans, a (α1→6)-mannopyranosyl chain linked to an acylated mannosyl-phosphoinositol in mycobacterial lipoglycans or a cysteine in lipopeptides/lipoproteins make the resulting molecules unique signatures of bacteria.

## Materials and Methods

### Lipoglycan purification – TLR2 ligands

DAG-based lipoglycans were purified as previously described from *M. luteus* (MlLM) [27], [28], *C. glutamicum* (CgLM) [30] and *S. mucilaginosus* (SmLM) [29]. MPI-based lipomannan was purified from *Mycobacterium smegmatis* (MsLM) [26]. Pam_3_CSK_4_, FSL-1 and SaLTA were purchased from InvivoGen. Lipoglycans, lipopeptides and LTA were deacylated by incubating with 2M NH_4_OH for 2h at 110°C.

### HEK-TLR2 experiments

The HEK-Blue™-2 cell line (InvivoGen), a derivative of HEK293 cells that stably expresses the human TLR2 and CD14 genes along with a NF-κB-inducible reporter system (secreted alkaline phosphatase) was used according to the manufacturer’s instruction. The different stimuli were added, at concentrations indicated in the figure legends, in 96-wells plates and cells were then distributed at 5×10^4^ cells per well in 200 µl DMEM culture medium (Lonza). Alkaline phosphatase activity was measured after 18 h by mixing 20 µl of the culture supernatant and 180 µl of Quanti-Blue™, and reading O.D. at 630 nm. To investigate the TLR dependence of stimuli activity, HEK-TLR2 cells were pre-incubated for 30 min at 37°C, before stimuli addition, with various antibodies: 5 µg/ml of monoclonal anti-TLR2 (clone T2.5, InvivoGen) or 10 µg/ml monoclonal anti-TLR1 (Clone H2G2, InvivoGen), monoclonal anti-TLR6 (Clone C5C8, InvivoGen) or an IgG1 isotype control (eBioscience).

### THP-1 experiments

THP-1-Dual™ cells (Invivogen), derivatives of THP-1 monocyte/macrophage human cells that stably express a NF-κB-inducible reporter system (secreted alkaline phosphatase), were used according to the manufacturer’s instruction. The different stimuli were added, at concentrations indicated in the figure legends, in 96-wells plates and cells were then distributed at 10^5^ cells per well in 200 µl RPMI 1640 culture medium (Lonza). After 18 h, NF-κB activation was measured as described above and cytokines were assayed in the culture supernatant by sandwich ELISA using commercially available kits (eBioscience). To investigate TLR2 dependence, cells were pre-incubated for 30 min at 37°C, before stimuli addition, with 10 µg/ml of anti-TLR2 monoclonal antibody (clone T2.5, InvivoGen) or isotype control (IgG1, eBioscience). For monitoring CD40 and MHC-II expression, cells were harvested and resuspended in Dubelco’s PBS, 0.5% BSA and labelled with CD40-PE or MHC-II-FITC conjugated antibody (Beckman Coulter). Cells were subjected to flow cytometry analysis by using the CellQuest software on a flow cytometer (FACSCalibur; Becton Dickinson).

### Statistical analysis

Results are expressed as a mean ± SD and were analyzed using One-way analysis of variance followed by Tukey test to determine significant differences between samples.
